# Nutrition and Autism Spectrum Disorder: Between False Myths and Real Research-Based Opportunities

**DOI:** 10.3390/nu13062068

**Published:** 2021-06-17

**Authors:** Antonio Narzisi, Gabriele Masi, Enzo Grossi

**Affiliations:** 1 Department of Child Psychiatry and Psychopharmacology, IRCCS Stella Maris Foundation, 56018 Pisa, Italy; gabriele.masi@fsm.unipi.it; 2 Villa Santa Maria Foundation, Tavernerio, 22038 Como, Italy; enzo.grossi@bracco.com

Autism Spectrum Disorder (ASD) is a multicomplex disorder characterized by an umbrella of specific issues in the areas of social communication, restricted interests, and repetitive behaviors [1]. The incidence of ASD is worldwide, and recent epidemiological data estimated it to be higher than 1/100 [2]. Promising evidence-based interventions for core symptoms in children and adolescent have been developed in recent years [3,4]. However, in addition to the core symptoms, ASD may have strong associations with other disorders and/or be associated with a plethora of behaviors and symptoms, such as those related to food selectivity and the consequent inadequate dietary intake [5].

Although some types of eating disturbances, such as food refusal, are also frequent in the general pediatric population, their prevalence appears to be significantly higher in ASD children, with rates ranging from 51% to 89% [6].

There is evidence that children with ASD consume fewer fruits and vegetables and have a lower intake of calcium and protein, compared to their typically developing peers [7]. Moreover, children with ASD prefer foods with high carbohydrate, content such as white bread, pizza, cakes, cookies, ice-cream, or “fatty” foods [8]. These foods are generally sweet, while on the contrary, bitter or sour tastes are more frequently rejected. This preference can cause a rise of blood glucose and triglycerides, resulting in overweight status and obesity [9], or endocrine disturbances such as diabetes [10], not to mention dental caries, since correct and constant oral hygiene is not always possible [11]. Additionally, children with ASD often have an inadequate intake of vitamin D, vitamin B12, vitamin C, calcium, zinc, and a lower consumption of dairies if compared with typically developed children [12]. This means that food selectivity is not just a matter of taste, but it is, most of all, a matter of health [13]. Finally, children with ASD, because of false myths, sometimes undergo to non-intentional and dangerous dietary restrictions protocols (e.g., casein and/or gluten-free) based on non-evidence-based attempt to improve behavioral disturbances or gastrointestinal symptoms [14].

Although a thorough empirical definition of food selectivity includes a high prevalence of food refusal and limited food repertoire choices as part of the child’s regular diet [5], the exact etiology of this aspect of ASD is not yet understood [15]. Family eating behavior and habits can obviously affect atypical food intake [15]. In fact, families who follow highly restricted diets generally have children with more restrictive eating behavior [13]. However, atypical eating behavior in ASD needs a better understanding and specific explanations.

Food selectivity can be considered an additional expression of the repetitive and restricted behaviors, which is part of the ASD phenomenology, resulting in a restricted variety of accepted foods [8,16]. In Diagnostic and Statistical Manual of Mental Disorders–Fifth Edition [1], oral fixation for the same kind of food is considered a manifestation of the insistence on sameness, inflexible adherence to routines or ritualized patterns of verbal or nonverbal behavior. The study of Suarez [16] showed a positive correlation between restricted and repetitive behaviors, measured through the Repetitive Behaviors Scale-Revised (RBS-R) and severity of food selectivity.

Food selectivity is often based on taste, texture, and presentation, and this issue may be related to the sensory over-responsivity, a sensory-processing disorder, expressed by an extreme over-reaction to sensations from any of the sensory system components: tactile, vestibular (i.e., sense of balance and spatial orientation), auditory, proprioceptive, gustatory, olfactory, and visual [17]. The hypothesis that sensory-over reaction may contribute to hypersensitivity to food textures and thus resulting in food selectivity has been widely reported [8,16]. According to a recent review by Page [6], there is clear evidence that impaired sensory processing is positively associated with feeding difficulties in children with ASD. Altered sensory perception appears to also be positively associated with food neophobia, but current evidence is from two small studies and thus warrants further investigation [18,19].

A block of scientific contributors have studied the link between the gut microbiota and ASD [20]. Most studies have shown that children with ASD have an altered gut microbiota, but, at the moment, it is impossible to properly compare these studies, based on very disparate case histories and methods. For example, an increase in Bacteroidetes and a corresponding decrease in Firmicutes were found in ASD subjects in 2 studies [21,22] when taken from the stool, but the relationship is reversed when dosed in the ileal mucosa [23]. Clostridia are increased in 2 studies [22,24] and a significant reduction in Bifidobacterial was shown in two studies [25,26] while Sutterella, a germ never previously detected in the human intestine, was found to be increased in both feces and ileal mucosa in 2 independent studies [23,27].

Further studies recently approached the link between ASD and nutrition, exploring the role of SGS (Sulforaphane-Glucosinolate) [28] and the potential benefits of vitamin D3 supplementation [29].

Dietary sulforaphane, well-known for its safety and lack of toxicity, has been explored for its possible capacity to reverse abnormalities, that have been hypothesized to be associated with ASD, including oxidative stress and lower antioxidant capacity, depressed glutathione synthesis, reduced mitochondrial function and oxidative phosphorylation, increased lipid peroxidation, and neuro-inflammation. Singh and colleagues [28] conducted a placebo-controlled, double-blind, randomized trial, involving adolescents and adults with moderate to severe ASD, who received the phytochemical sulforaphane—derived from broccoli sprout extracts—or indistinguishable placebo. The effects on behavior of daily oral doses of sulforaphane for 18 wks., followed by 4 wks. without treatment, were assessed with the Aberrant Behavior Checklist (ABC), the Social Responsiveness Scale (SRS), and the Clinical Global Impression Improvement Scale (CGI-I Guy), completed by parents/caregivers and physicians. Participants receiving placebo experienced minimal change, whereas those receiving sulforaphane showed substantial improvements, 34% according to ABC, and 17% according to SRS. Based on CGI-I, a significantly greater number of participants receiving sulforaphane had improvement in social interaction, abnormal behavior, and verbal communication. Upon discontinuation of sulforaphane, total scores on all scales worsened to pretreatment levels.

Regarding possible benefits of vitamin D3 supplementation, Grossi and colleagues [30] underlined that very narrow spectrum of habits in persons with ASD may easily predispose to nutritional deficiencies, namely vegetables, both cooked and raw (tomatoes in particular), and fish (the main source of vitamin D3). Three randomized controlled trials (RCTs) [31,32,33] included in a recent meta-analysis by Bingbing and colleagues [34] suggested that vitamin D supplementation was beneficial for improving symptoms in children with ASD, demonstrated by the significantly lower SRS and Childhood Autism Rating Scale (CARS) scores.

In conclusion, to date, there is a lack of sufficiently robust evidence to support specific dietary interventions in children with ASD, although some small, documented real research-based opportunities (e.g., vitamin D3; SGS) may open interesting glimmers. It is, therefore necessary to increase basic research on ASD, which could provide useful suggestions on nutritional interventions. In all the families with a child/adolescent with ASD, and even more in those presenting specific food selectivity, a close monitoring of correct eating habits by family pediatricians is warranted, with specific diagnostic procedures to prevent health problems, and possibly adopting evidence-based approaches when needed (e.g., psychoeducation and/or cognitive behavioral methods) [35,36].
